# Maintenance Therapy Post-Stem Cell Transplantation for Patients with T-Cell Lymphomas

**DOI:** 10.1007/s11899-024-00743-w

**Published:** 2024-10-19

**Authors:** Zachary Braunstein, Jonathan E. Brammer

**Affiliations:** Division of Hematology, Department of Internal Medicine, James Comprehensive Cancer Center, The Ohio State University Wexner Medical Center, 2121 Kenny Road, Room 7168, Columbus, OH 43210 USA

**Keywords:** T-cell lymphoma, Peripheral T-cell lymphoma, Stem cell transplantation, Bone marrow transplantation, Allogeneic, Autologous, Therapeutics, Maintenance therapy

## Abstract

**Purpose of Review:**

Given the poor outcomes for peripheral T-cell lymphomas (PTCL), stem cell transplant (SCT) remains an important therapeutic approach. Post-SCT relapse is common and maintenance therapy post-SCT is increasingly being utilized. Here we review the use of post-SCT maintenance therapy for PTCL patients.

**Recent Findings:**

Maintenance therapy is increasingly utilized to decrease post-SCT relapse and improve outcomes in PTCL. Ongoing and completed post-SCT maintenance trials utilizing agents such as romidepsin, brentuximab vedotin, duvelisib, and pembrolizumab have shown efficacy in decreasing relapse. Further, additional agents with efficacy in PTCL have emerged that may inform future maintenance approaches.

**Summary:**

Maintenance therapy is a promising approach to maintain response after SCT in PTCL. While several trials are ongoing to evaluate maintenance therapy in PTCL, current data suggests this may be an effective method to decrease post-SCT relapse.

## Introduction

Peripheral T-Cell lymphomas (PTCLs) are a heterogeneous group of non-Hodgkin lymphoma (NHL) with an aggressive disease course that constitute approximately 10-15% of NHL in western countries [1–3]. There are more than 25 unique types of PTCL, and in North America the most common subtypes are Peripheral T-Cell Lymphoma – Not Otherwise Specified (PTCL-NOS) (34%), Angioimmunoblastic T-Cell Lymphoma (AITL) (16%), anaplastic lymphoma kinase (ALK) negative Anaplastic Large Cell Lymphoma (ALCL) (16%), and ALK positive ALCL (8%). While standard frontline therapeutic approaches utilizing cyclophosphamide/doxorubicin/vincristine/prednisone (CHOP)-based chemotherapy regimens can induce long-term remissions [3, 4], relapse remains the primary cause of long-term treatment failure, and 5-year overall survival (OS) for PTCL is poor at 30-35% [5–7]. Even for patients with ALCL, whose outcomes are better than CD30-negative PTCL due to the ability to target CD30 with brentuximab vedotin, long-term data demonstrates increased risk of relapse at 5-years [8, 9]. For patients that do relapse, outcomes are poor, and without stem cell transplantation (SCT) the median OS is only 4 months [10–12].

Given the poor outcomes with standard therapy, SCT, including both autologous (auto-SCT) and allogeneic (allo-SCT) transplant, remains an important treatment modality to improve survival in first remission, or provide curative therapy for patients with relapsed/refractory (r/r) PTCL [5, 13]. Improvement in SCT outcomes in PTCL are crucially important, since novel cellular therapies such as chimeric antigen receptor (CAR) T-cells face significant logistical and therapeutic challenges in PTCL, and are currently unavailable for this disease. Auto-SCT typically is utilized to consolidate remissions in first complete remission (CR1), or second complete remission (CR2) in select cases, while allo-SCT is typically utilized with curative intent in fit patients what attain a 2^nd^ CR/PR in the setting of relapsed PTCL, or those with high-risk subtypes. Despite SCT, relapse remains the primary cause of treatment failure post-SCT.

In recent years, the use of maintenance therapy, whereby novel therapeutics are applied in the post-SCT setting to decrease relapse have demonstrated success in patients with hematologic malignancies, particularly Hodgkin lymphoma and multiple myeloma [8, 14–16]. As several promising agents have become available for patients with PTCL over the past decade, including romidepsin [17], belinostat [18], brentuximab vedotin (BV) [8], valemetostat [19], duvelisib [20], and pembrolizumab [21], these drugs are increasingly being applied in the post-SCT setting as maintenance with the goal to decrease relapse. Importantly, the eligibility of SCT for patients with PTCL is limited to those who can attain a remission, and many patients ultimately cannot receive the benefit of SCT due to progressive disease. Maintenance therapy post-SCT is particularly attractive in patients with r/r PTCL, for whom CR is difficult to attain, as it has to the potential to increase patient eligibility for curative SCT in those who cannot attain a full CR pre-SCT. In this review, we will evaluate current and recently completed maintenance approaches for patients with PTCL and consider potential promising future therapeutic approaches to maintenance.

### SCT in PTCL Overview

The use of SCT and associated outcomes has been summarized in our recent review [5]. For eligible patients, consolidative auto-SCT, in CR1 remains standard of care for diseases other than ALK++ ALCL with low IPI score (IPI 0-1). Improvement in PFS in this population has been observed in several retrospective studies, with an approximate decrease in relapse risk of about 10-15% in patients receiving consolidative auto-SCT [5, 22, 23]. Even with the addition of BV frontline for patients with ALCL, consolidative auto-SCT still appears to improve long-term outcomes, though additional data is needed [24]. Despite the increased probability of cure with consolidative auto-SCT, PFS in these patients post-auto-SCT still is only approximately 50%, with the majority of failures due to relapse [23, 25]. Allo-SCT is not utilized as frontline consolidation therapy, with the exception of rare PTCL subtypes (e.g. hepatosplenic T-cell lymphoma (HSTCL), monomorphic epitheliotropic intestinal T-cell lymphoma (MEITL) [26–28].

In the relapsed/refractory (r/r) setting, outside of SCT, there are no curative therapies for PTCL. For patients that have relapsed disease, salvage therapy can be attempted with treatments such as romidepsin, bendamustine, BV, belinostat, ifosfamide/carboplatin/etoposide (ICE), and duvelisib, among others [12], with the goal of achieving enough of a CR/PR needed proceed with SCT. Auto-SCT is associated with a cure in only about 33% of patients with r/r PTCL in CR2+, and is reserved for select patients ineligible for allo-SCT, but eligible for auto-SCT [23]. Auto-SCT can be considered in CR2+ for patients with relapsed ALCL if they did not receive consolidative auto-SCT in CR1 [22, 23]. Allo-SCT can provide curative therapy in r/r PTCL, but even after allo-SCT, there is almost a 50% relapse rate at 5 years with non-relapse mortality (NRM) rates ranging from 12%-46% at 5 years and OS 57% at 5 years [29–32]. These results only represent patients that are able to obtain a remission (CR/PR), and do not represent the majority of patients that are not able to obtain a remission. Since SCT is the only curative mechanism for patients with relapsed/refractory PTCL, it is imperative to develop innovative therapeutic strategies that can facilitate a higher response rate for patients with PTCL. Further, it is crucial to improve post-SCT relapse mitigation strategies to enable a larger proportion of patients to proceed with SCT with curative intent, including those who cannot attain a CR.

### Role of Maintenance in PTCL

Current approaches to maintenance therapy post-SCT (allo-SCT and auto-SCT) can be classified based upon two primary treatment goals: 1) To decrease post-SCT relapse in patients entering SCT in CR and 2) increase the proportion of patients eligible for curative-SCT who cannot attain a CR, as a majority of patients with r/r PTCL are unable to attain a CR prior to SCT. Broadly, maintenance approaches can be divided into two separate approaches to attain these goals: direct anti-tumor effect, and immune-modulation. In this review we highlight existing data for individual maintenance therapies post-SCT (allo-SCT and auto-SCT) for PTCL and provide evidence-based recommendations for emerging novel therapies for post-transplant maintenance. A summary of maintenance therapies can be seen in Table 1.

**Table 1:** Previous and Current maintenance trials in PTCL

Treatment	Number of Patients (*n*)	Cytotoxic or Immunomodulation	Type of transplant	Conditioning	Trail Phase	Dosing	Survival
Romidepsin [33]	34	Cytotoxic*	Autologous	BEAM	II	14 mg/m^2^ weekly**	2-year PFS: 62%
Romidepsin [34]	18 (9 with TCL)	Dual*	Allogeneic	Flu/Bu	I/II	8 mg/m^2^ every 2 weeks	3-year PFS: 41%
Duvelisib [35]	12 (9 with TCL)	Cytotoxic	Autologous	Not Specified	II	25 mg BID***	Not Reported
Brentuximab Vedotin [36]	43 (26 had transplant)	Cytotoxic	Autologous	Not Specified	II	1.8 mg/kg every 3 weeks for 10 cycles	2-year PFS: 80% (in transplanted)
Pembrolizumab [37]	21	Immunomodulation	Autologous	BEAM	II	200 mg every 3 weeks for 8 doses	18 month OS: 94%

BEAM: Carmustine, Etoposide, Cytarabine, Melphalan; BID: two times per day; B-NHL: B-cell Non-Hodgkin lymphoma; Bu: Busulfan; EBV: Epstein-Barr Virus; ENKTL: Extranodal NK/T-Cell Lymphoma; Flu: Fludarabine; kg: kilogram; mg: milligrams; OS: Overall Survival; PFS: progression free survival; p-IFN: Pegylated interferon; TCL: T-Cell lymphoma

*Romidepsin can be either Cytotoxic or Immunomodulatory depending on the approach/study

**At 6 months dosing was switched to every 3 weeks and at 1 year dosing was changed to monthly for a total of 2 years maintenance

***If CR at Day +100 then dosing was switched to 14 days on/14 days off

## Romidepsin

### Romidepsin in Autologous SCT

Romidepsin is a histone deacetylase (HDAC) inhibitor that has been shown to induce durable responses in r/r PTCL [38–40]. Furthermore, romidepsin and HDAC inhibitors have demonstrated potentially immune-modulatory effects to decrease GVHD while potentially enhancing the graft-versus-lymphoma effect [41–43]. Khan *et al.* performed a phase II open label trial where patients received an auto-SCT with carmustine/etoposide/cytarabine/melphalan (BEAM) conditioning followed by maintenance romidepsin for T-cell lymphoma [33]. Romidepsin 14 mg/m2 was started between 42-80 days post auto-SCT on an every other week schedule for the first six months of treatment. This was followed by every three weeks dosing through one year and every four weeks through the end of two years post auto-SCT. 47 patients were consented to trial and 34 patients received romidepsin, 26 (76%) of which were transplanted in CR1/PR1, and 5 (15%) of which were transplanted in CR2/PR2, and 2 of which were transplanted with high risk histologies. Of the 34 patients that received romidepsin, 5 patients required a dose reduction due to toxicity though additional data on dose reductions is unavailable. The 2-year PFS of 62% across all patients did not meet the desired primary endpoint of PFS, which was 70% at 2-years, though did show romidepsin to be safe and tolerable with the most common side effects being fatigue (73%), thrombocytopenia (48%), and anemia (48%). Further, while full results are not available for this study, the reported results suggests a potential PFS improvement at 2 years compared to historical controls. Lack of additional published data prevents the use of this potentially promising approach post-auto-SCT in PTCL.

## Romidepsin in Allogeneic SCT

Hosing *et al.* recently completed a phase I/II clinical trial evaluating maintenance romidepsin (m-Rom) after allo-SCT with either myeloablative or reduced intensity fludarabine/busulfan conditioning in patients with T-cell malignancies (including T-cell leukemias and lymphomas) [34]. This study also included a phase I portion, whereby low-dose romidepsin was added to conditioning chemotherapy as a synergistic agent. Patients initiated m-Rom between days +28 and +100 and continued through 1-year post allo-SCT with a second optional year. Patients were permitted to receive romidepsin as a treatment prior to transplant. Dosing was started at 8 mg/m^2^ every 2 weeks with dose reductions to 4mg/m^2^ and 2mg/m^2^ for toxicities. Of 28 patients treated, 46% (*n*=13) had PTCL, while 54% (*n*=15) had T-cell leukemia. 18 patients (including 9 with PTCL, 62%) received m-Rom with a median of 13 cycles (range: 1-47). The median OS was 3.3 years and median PFS was 2.3 years. Full results have not yet been reported, but are promising, with one-year cumulative incidence of relapse (across subtypes) of 18%, and three-year PFS rates of 61% and 41% respectively. The trial met its pre-specified endpoint of 20% decreased cumulative incidence of relapse at 1-year; dramatically lower than the expected rate of relapse for all disease (18% vs 55%). Impressively, the cumulative incidence of relapse (CIR) for PTCL patients at 1 and 3-years was 10%, and no patients with PTCL relapsed (1 of 2 patients with CTCL relapsed). Eight patients had grade 3/4 AE and one patient discontinued due to toxicity (although unlikely related to m-rom). The authors presented evidence of enhancement of the graft-vs-lymphoma effect by demonstrating significantly enhanced NK-cytotoxicity in the patients receiving m-Rom compared to patients that did not receive m-Rom. While final publication and longer follow-up of these results is needed, m-Rom is a promising therapy for patients receiving allo-SCT for PTCL (and T-cell leukemias), and should be considered for patients post allo-SCT with PTCL; particularly those not entering SCT in a CR.

## Duvelisib

Duvelisib is a potent phosphoinositide-3-kinase delta (PI3K-δ) and gamma (PI3K-γ) inhibitor that has been shown to be effective in PTCL and b-NHL via tumor microenvironment disruption and inhibition of malignant cell proliferation and differentiation [44, 45]. Saba *et al.* performed a phase II trial to evaluate the safety and efficacy of duvelisib maintenance post auto-SCT for PTCL and B-NHL [35]. 12 patients were enrolled on trial, including seven PTCL patients (1 PTCL-NOS, 3 ALCL, and 3 AITL). Patients were permitted to receive duvelisib as a treatment prior to transplant. At the time of SCT 11 patients were in a CR and one patient was in a PR. No data was provided regarding conditioning regimen for auto-SCT or CR status (e.g. CR1, CR2, etc). Patients initiated duvelisib 25mg twice daily (BID) approximately 30 days after auto-SCT. If the patient was in a CR at day +100 they were changed to 25mg BID for 14 days on alternating with 14 days off. Patients with PR were maintained on continuous duvelisib while those with persistent or progressive disease were taken off trial. Median follow-up time was 24 months and the median duration of duvelisib maintenance was 8.7 months. Four patients relapsed (33%), including two of seven (29%) PTCL patients, and all patients remain alive at the time or reporting. The most common side effects were cytopenias, nausea/vomiting/diarrhea, and transaminitis. Grade 2-3 adverse events (AE) were seen in 6 patients (two with diarrhea, two with neutropenia, one with transaminitis, and one with lymphopenia). Permanent discontinuation of treatment occurred in three patients due to grade 3 toxicities (transaminitis, lower extremity muscle weakness, and diarrhea). Toxicities significantly improved when patients were changed to 14 days on/14 days off instead of 28 days continuous. Based on this preliminary data maintenance duvelisib appears to be safe and tolerated at a 14 days on/14 days off schedule. Greater numbers of PTCL patients are needed to evaluate the efficacy of duvelisib for maintenance after auto-SCT for PTCL.

## Brentuximab Vedotin

BV is an anti-CD30 antibody drug conjugate that is utilized in the treatment of CD30+ PTCL with improvement in PFS and OS [8]. In the AETHERA trial in Hodgkin Lymphoma patients, maintenance therapy post-auto-SCT with BV showed a significant improvement in PFS, exceeding 80% at 3-years [46]. While it was shown in the ECHELON-2 study that the addition of BV to frontline treatment in CD30+ ALCL improved survival over CHOP, the utility of BV consolidation or maintenance post-SCT is not known. To address this question, Herrera *et al.* performed a phase II study to evaluate the addition of etoposide to BV-CHP (BV-CHEP) with BV consolidation (without auto-SCT) or maintenance after auto-SCT [36, 47]. Patients received six cycles of BV-CHEP and those with a response could receive up to 10 cycles of BV maintenance dosed at 1.8 mg/kg every 3 weeks after auto-SCT. Patients ineligible or unwilling to proceed with auto-SCT had the option to complete maintenance BV alone. 43 patients completed BV-CHEP and 26 (54%) proceeded to auto-SCT. Twenty-one patients went on to receive BV maintenance post auto-SCT and the landmark 2-year PFS from end of BV-CHEP in responding pts (*n*=41) was 80% in pts who underwent auto-SCT vs 47% in pts who did not (*p*=0.05). While these results are encouraging, SCT vs no-SCT evaluations are subject to selection bias, and results will need to be confirmed in the expanded cohort evaluating non-ALCL CD30+ patients in this currently ongoing trial (NCT03113500). While broad use of maintenance BV post-SCT cannot yet be recommended, the use of maintenance BV in patients with confirmed or impending relapse post-SCT is supported by available evidence.

## Pembrolizumab

Pembrolizumab is a programmed cell death protein 1 (PD-1) inhibitor that has been shown to have activity in r/r PTCL, especially with the increased PD-1expression in a variety of NK/T-cell lymphomas by malignant T cells [21]. Merrill *et al.* recently completed a phase II study to evaluate the use of pembrolizumab after auto-SCT in patients with PTCL [37]. 21 patients were enrolled in the trial and 19 (90%) had a CR1 at the time of auto-SCT and 20 (95%) had a CR1 at the study entry. Patients had to be in first remission (CR or PR) in order to enroll in the study. Patients were excluded if they had central nervous system (CNS) involvement or if they had received previous immunotherapy with anti-PD-1 or anti- cytotoxic T-lymphocyte associated protein 4 (CTLA-4) treatment. Patients received 200mg IV every 3 weeks for up to eight doses and were recommended to start within 21 days post-transplant but could initiate up to 60 days post-SCT per protocol. A majority of patients (90%) had conditioning with BEAM, while the others had either busulfan/cyclophosphamide/thiotepa (5%) or the conditioning regimen was unknown (5%). Of the 21 patients enrolled in the trial, *n*=9 (43%) had stage IV disease and *n*=14 (67%) completed all eight cycles. The most common reason for discontinuation was progression, in four of the seven patients that did not complete all eight cycles. The 18-month OS and PFS were 94.4% and 83.6%, respectively. There was no statistically significant difference between subtypes, although interestingly none of the patients with Epstein-Barr virus (EBV) positive extranodal natural killer/T-cell lymphoma (ENKTL) (*n*=3) progressed with maintenance therapy. 81% (17/21) of patients had toxicity including five (24%) with grade 3/4 AE, the most common of which were gastrointestinal in nature with transaminitis, abdominal pain, diarrhea, and colitis. There were 14 immunotherapy related AEs (irAEs) in the study, that affected 7 of 21 (33%) of patients, with four grade 2 events and four grade 3 events. The most common grade ≥2 irAEs being transaminitis (*n*=3; 15%,) hypothyroidism (*n*=2; 10%), and diarrhea (*n*=2; 10%). While no instances were observed in this trial, there is concern that PD-1 inhibitors can induce hyper-progression in PTCL [48]. This first observed in a trial evaluating nivolumab in which treatment was terminated early due to the development hyper-progression in 33% of patients [49]. The use of checkpoint inhibitors post-SCT is limited to auto-SCT, due to increased risk of GVHD post-allo-SCT. While longer-term follow-up is needed, the results of maintenance nivolumab post-SCT is a promising approach that should be considered in patients with PTCL.

### Future Maintenance Approaches

With the high rates of relapse post-SCT in PTCL, and despite promising emerging data on maintenance regimens post-SCT, further investigation is needed to improve survival outcomes in this high-risk patient population. In particular, maintenance approaches could best improve outcomes, and broaden patient eligibility for curative therapy by targeting two key populations: those receiving auto-SCT without a CR/PR and those ineligible for allo-SCT in CR2+ yet eligible for auto-SCT. Many patients are unable to proceed with an allo-SCT due a variety of reasons such as comorbidities, age, etc. and with the aggressive nature of these diseases, maintenance therapy post-SCT may be the only way to maintain response in these patients. Maintenance also has the potential to improve post-allo-SCT outcomes by decreasing relapse. Below we will discuss several potential therapeutic approaches for maintenance therapy post-SCT. A summary of these approaches can be found in Table 2.

**Table 2:** Potential Novel Maintenance Approaches

Treatment	Cytotoxic or Immunomodulation	Disease	Regimen	TCL Response Rates	Special Considerations
Azacitidine [50]	Cytotoxic	Treatment naїve PTCL	300 mg daily for 7 days before cycle 1 of CHOP and 14 days before cycles 26;	2-year OS/PFS: 68.4/65.8%	Improved responses in those with TFH phenotype and/ or *TET2*, IDH ½, or *DNMT3A* mutations
Azacitidine [51]	Cytotoxic	AITL/TFH PTCL	300 mg BID for 14 days of a 28 day cycle	3-month ORR: 33%	None
Ruxolitinib [52]	Dual	r/r PTCL	20 mg BID	ORR: 25%	Increased ORR in those with JAK/STAT mutation
Valemetostat [53]	Cytotoxic	r/r TCL	200 mg daily	ORR: 44%	None

AITL: Angioimmunoblastic TCL; BID: two times per day; CHOP: cyclophosphamide, doxorubicin, vincristine, prednisone; MF: Mycosis fungoides; ORR: Overall response rate; OS: Overall survival; PFS: progression free suvivsl; PTCL: Peripheral T-cell lymphoma; r/r: Relapsed/refractory; TCL: T-Cell Lymphoma; TFH: T follicular helper

## Azacitidine

Azacitidine is a hypomethylating agent (HMA) that has been approved for myelodysplastic syndrome (MDS). It has been shown that many PTCLs with a T-follicular helper (TFH) phenotype, including AITL, express mutations in *TET2, DNMT3A, RHOA,* and/or *IDH1/2* [54, 55] and in MDS, these mutations can predict a response to HMAs [56], Ruan *et al* recently performed a phase II study of oral azacitidine (CC-486) plus CHOP as initial treatment for PTCL [50]. CC-486 300mg daily was given for 7 days before C1 of CHOP and then 14 days before C2-6 of CHOP. The 2-year PFS/OS was 65.8%/68.4% for the entire cohort and 69.2%/76.1% for PTCL-TFH. Patients with a *TET2* mutation had a significantly improved rate of CR and PFS and patients with a DNMT3A mutation had a worse PFS. This regimen is currently being investigated in a phase II trial (NCT 04803201). Further, the phase III Oracle study tested single-agent oral 5-azacytidine in 86 patients with AITL and TFH PTCL, and demonstrated an ORR 33% (12% CR, 21% PR) [51]. Based off of these trials, azacitidine is a potential option for maintenance therapy for patients with PTCL post-SCT, especially in patients that have a TFH phenotype with characteristic mutations.

## Ruxolitinib

The Janus kinase (JAK)-signal transducer and activator of transcription (STAT) pathway has been recognized as a major component in PTCL with activating mutations in JAK1, 2, and 3 and STAT3 and 5b in multiple different T-Cell malignancies [57–60]. Moskowitz *et al* recently completed a phase II study evaluating the JAK 1/2 inhibitor, ruxolitinib, for patients with r/r PTCL or mycosis fungoides (MF) [52]. Patients received ruxolitinib 20mg twice per day until progression with an ORR of 25% (6% CR, 19% PR). Adverse effects primarily involved cytopenias which is a known side effect of ruxolitinib. Importantly, ruxolitinib is effective at preventing graft-versus-host disease, and could provide dual benefits in PTCL, similar to what has been observed in myelofibrosis [61, 62]. Anecdotally, we have utilized this approach in patients with relapsed PTCL post-allo-SCT, with and without GVHD with improvement in GVHD and/or induced remission in patients with low-level (not florid) disease relapse. Based off of this study, and our experience, our center is leading a national effort evaluating ruxolitinib post auto-SCT and allo-SCT to decrease relapse.

## Valemetostat

Valemetostat is an enhancer of zeste homolog (EZH)1 and EZH2 that has shown activity in patients with r/r PTCL [19]. In the Phase II Valentine-PTCL01 study patients with r/r PTCL received Valemetostat 200mg per day in continuous 28-day cycles until disease progression or toxicity [53]. This trial demonstrated a promising ORR at 44% (14% CR, 29% PR) with a median DOR of 11.9 months. Valemetostat was well tolerated with the most common adverse effects including cytopenias. Based on these studies it would be a promising approach to utilize valemetostat as a maintenance treatment for patients that receive a SCT for PTCL.

### Recommendations for Utilization of Maintenance

While data continues to emerge regarding the utilization of maintenance therapies post-SCT in patients with T-cell lymphoma, existing data support the use of maintenance therapies in this group of patients. Maintenance therapies are likely most effective in patients entering SCT without a CR, given higher relapse rates. Our clinical practice is to utilize m-Rom for all patients with PTCL or CTCL after allo-SCT for the first year post-SCT; particularly in patients entering allo-SCT without a CR. For patients with concomitant graft-versus-host disease (GVHD) post-allo-SCT and concerns for relapse, we utilize ruxolitinib maintenance, as ruxolitinib can treat both the GVHD and PTCL, or prevent PTCL in the absence of relapsed disease. For allo-SCT patients without GVHD, who have not started maintenance (or elected not to), with evidence of relapse, we will initiate m-Rom for ~1 year post allo-SCT. Continuation of maintenance for more than 1 year post-allo-SCT is evaluated on a case-by-case basis, though we strongly recommend maintenance therapy in those patients for whom evidence of disease remains. For auto-SCT patients, maintenance at our institution is not standard, though with recent data utilizing pembrolizumab post-SCT, we would strongly consider utilizing this agent for those entering auto-SCT without a CR.

## Conclusions

Patients with PTCL have a high rate of progression or relapse, even after standard of care treatment both with or without SCT and the outcomes for r/r disease are extremely poor. Post-SCT maintenance therapy is a promising approach to maintain remission and improve survival outcomes in patients with both auto-SCT and allo-SCT. Maintenance approaches are particularly important for those who cannot attain a remission prior to SCT, or those ineligible for curative therapy with allo-SCT who can receive auto-SCT. Of the maintenance approaches studied to date, maintenance romidepsin (M-rom) post allo-SCT and maintenance pembrolizumab post auto-SCT provide the most complete results, and should be considered in patients receiving SCT. With several trials ongoing to evaluate maintenance therapy in TCL, data to date suggest this may be an effective method to decrease relapse in patients post-allo-SCT and auto- SCT and warrants further investigation.

## Key References

- Brammer JE, Zinzani PL, Zain J, et al. Duvelisib in Patients with Relapsed/Refractory Peripheral T-Cell Lymphoma from the Phase 2 Primo Trial: Results of an Interim Analysis. Blood. 2021;138:2456.
  - This study illustrates new data on the use of Duvelisib as a novel agent for PTCL
- Savage KJ, Horwitz SM, Advani R, et al. Role of stem cell transplant in CD30+ PTCL following frontline brentuximab vedotin plus CHP or CHOP in ECHELON-2. Blood Adv. 2022;6(19):5550-5555.
  - This paper illustrates the importance of consolidative stem cell transplant in CD30+ PTCL patients that achieve a CR following treatment with A+CHP.
- Hamadani M, Ngoya M, Sureda A, et al. Outcome of allogeneic transplantation for mature T-cell lymphomas: impact of donor source and disease characteristics. Blood Adv. 2022;6(3):920-930.
  - This study illustrates that allogeneic transplantation can provide a durable PFS in patients with PTCL.
- Khan N, Khimani F, Shustov AR, et al. Update of a phase II, multicenter study of high-dose chemotherapy with autologous stem cell transplant followed by maintenance romidepsin for T-cell lymphoma. Journal of Clinical Oncology. 2021;39(15).
  - This study shows the maintenance trial data for Romidepsin after autologous stem cell transplant.
- Hosing C, Braunstein Z, McLaughlin E, et al. Post-Allograft Romidepsin Maintenance Mitigates Relapse Risk and Stimulates the Graft-Versus-Malignancy Effect through Enhanced NK-Cell Cytotoxicity in Patients with T-Cell Malignancies: Final Results of a Phase I/II Trial. Blood. 2023;142(Supplement 1):184.
  - This study shows the maintenance trial data for Romidepsin after allogeneic stem cell transplant.
- Saba R, Mehta-Shah N, Ghobadi a, DiPersio JF, F. CA, We N. Phase II Trial of Duvelisib Maintenance after Autologous Stem Cell Transplant in T-Cell and B-Cell Non-Hodgkin Lymphomas: Results of Safety Lead in. Blood. 2023;142(Supplement 1):6245.
  - This study shows the maintenance trial data for Duvelisib
- Herrera AF, Zain J, Savage KJ, Feldman T, Brammer JE, Chen L, Puverel S, Popplewell L, Budde LE, Mei M, Hosing C, Nair R, Leslie L, Daniels S, Peters L, Forman S, Rosen S, Kwak L, Iyer SP. Brentuximab vedotin plus cyclophosphamide, doxorubicin, etoposide, and prednisone followed by brentuximab vedotin consolidation in CD30-positive peripheral T-cell lymphomas: a multicentre, single-arm, phase 2 study. Lancet Haematol. 2024;11(9):e671-e681.
  - This study shows the maintenance trial data for Brentuximab Vedotin
- Merrill MH, Dahi PB, Redd RA, et al. A phase 2 study of pembrolizumab after autologous stem cell transplantation in patients with T-cell non-Hodgkin lymphoma. Blood. 2023;142(7):621-628.
  - This study shows the maintenance trial data for Pembrolizumab
- Ruan J, Moskowitz A, Mehta-Shah N, et al. Multicenter phase 2 study of oral azacitidine (CC-486) plus CHOP as initial treatment for PTCL. Blood. 2023;141(18):2194-2205.
  - This study shows the efficacy of Azacitidine as a novel agent in patients with relapsed or refractory PTCL.
- Dupuis J, Tsukasaki K, Bachy E, et al. Oral Azacytidine in Patients with Relapsed/Refractory Angioimmunoblastic T-Cell Lymphoma: Final Analysis of the Oracle Phase III Study. Blood. 2022;140(Supplement 1):2310-2312.
  - This study shows the efficacy of Azacitadine as a novel agent in patients with relapsed or refractory PTCL.
- Moskowitz AJ, Ghione P, Jacobsen E, et al. A phase 2 biomarker-driven study of ruxolitinib demonstrates effectiveness of JAK/STAT targeting in T-cell lymphomas. Blood. 2021;138(26):2828-2837.
  - This study shows the efficacy of Ruxolitinib as a novel agent in patients with relapsed or refractory PTCL.
- Horwitz SM, Izutsu K, Mehta-Shah N, et al. Efficacy and Safety of Valemetostat Monotherapy in Patients with Relapsed or Refractory Peripheral T-Cell Lymphomas: Primary Results of the Phase 2 VALENTINE-PTCL01 Study. Blood. 2023;142(Supplement 1):302.
  - This study shows the efficacy of Valemetostat as a novel agent in patients with relapsed or refractory PTCL.

## Data Availability

No datasets were generated or analysed during the current study.
